# Control System Development and Implementation of a CNC Laser Engraver for Environmental Use with Remote Imaging

**DOI:** 10.1155/2022/9140156

**Published:** 2022-09-10

**Authors:** Hani Attar, Amer Tahseen Abu-Jassar, Ayman Amer, Vyacheslav Lyashenko, Vladyslav Yevsieiev, Mohammad R. Khosravi

**Affiliations:** ^1^Department of Energy Engineering, Zarqa University, Zarqa, Jordan; ^2^Faculty of Computer Science and Information Technology, Ajloun National University, Ajloun, Jordan; ^3^Department of Media Systems and Technology, Kharkiv National University of Radio Electronics, Kharkiv, Ukraine; ^4^Department of Computer-Integrated Technologies, Automation and Mechatronics, Kharkiv National University of Radio Electronics, Kharkiv, Ukraine; ^5^Department of Computer Engineering, Persian Gulf University, Bandar Bushehr, Iran

## Abstract

This article is aimed at studying the features of the control systems development for a small-sized Computer Numerical Control (CNC) portative laser engraver. The CNC is implemented in mobile maintenance and repair platforms for remote sensing of the environment where the wild environment may not allow us to access the animals and places. The proposed work in this paper is based on recent research, which shows that applying the automated CNC speeds up the processes of repair, modernizes the equipment size, and significantly reduces the economic costs; accordingly, the authors developed a block diagram of a portable CNC laser engraver. The choice of the hardware was also made, taking into account the possibility of quick replacement in the field, which reduces the repair time and the cost of the developed layout. A control system based on the selected modules was synthesized, and a stability check was carried out using MatLab tools. To check the correctness of the developed control system, the authors developed and assembled an experimental layout to illustrate the results of engraving on such a layout. Finally, the stability and sensitivity of the proposed system have been obtained and proved that the system works in a comfortable zone of stability. The obtained results show that the proposed CNC laser engraver has achieved the expected improvements (high speed, small size, short production and repairing time, minimum human influence factor, and achieving a better outcome).

## 1. Introduction

The introduction of modern progressive technologies in all spheres of human activity was presented in the concept of the fourth industrial revolution (Industry 4.0) [1–5]; resulting in making it possible to apply and integrate modern information technologies such as Artificial Intelligence (AI) [6, 7], Big Data (BD) [8, 9], and Internet of Things (IoT) with hardware solutions based on Microcontroller Unit (MCU) [10–14]. Accordingly, it becomes possible to automate the production process through the introduction of new control systems such as Computer Numerical Control (CNC) machines or Programmable Logic Controller (PLC) [15–17]. The implementation of CNC and PLC provides a wide opportunity to optimize the production process, improve the adaptation flexibility and customization for the release of new products, and minimize the influence of the human factor at the production stage. When combining the Computer-Aided Technologies (CAx) with CNC and PLC, the resulted symbiosis technique enables the implementation of the following solutions on the basis of CNC [18]:

- Additive 3D printing (plastic or metal)
- Industrial milling (metals, plastic, or wood)
- Laser engraving (metal, plastic, wood, etc.)

All of the above-mentioned solutions are regarded as an integral part of the Cyber-Physical Manufacturing Systems (CPPS) that are used in enterprises with flexible production cycles.

During the analysis of the CNC machines' performance, the authors gave good attention to the NATO reference book and the Logistics Handbook, mainly for Logistics in the Maintenance and Repair sections. Indeed, understanding the repairing field is regarded as a set of measures to restore the suitability of the material part for the operation in the shortest possible time [19]. Armament Repair Shop Set (ARSS) is a one-sided sliding shelter of a repair shop that contains small-sized portable CNC machines, on the basis of which you can quickly repair or upgrade the equipment without sending it to manufacturers [20, 21]. Similar portable machines can also be useful for field work and small workshops. At the same time, it should be noted that most of the CNC machines that are mainly designed for industrial applications have large overall dimensions and cannot be used in ARSS systems [22].

Thus, the development of a small-sized portative CNC laser engraver with high engraving accuracy is an actual and promising research area. Accordingly, to conduct such a study, it is necessary to solve the following tasks:

- Conduct research and analysis of the vectors of input parameters for a laser engraver to obtain a good description
- Establish the relationship between the input and output parameters for control system implementations
- Develop a structural control system for laser engraver
- Select hardware modules for the control system and executing devices, which rely on the selected hardware modules and synthesize the control system and the stability check that applies the theories of automatic control
- Assemble an experimental portable model of a laser engraver and conduct trial engravings based on the developed control system to confirm the main theoretical provisions of the proposed work and the performance of a portable laser engraver model

## 2. System Design and Implementation

Matching the selected CNC and the laser machine equipment's hardware is regarded as the backbone of the proposed work.

### 2.1. Match Selection of CNC Laser Machine Control Key Parameters and the Equipment's Hardware

**Figure 1:**
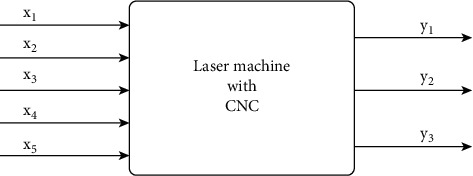
CNC laser machine as an object of control.

The brightness of images taken with the help of a laser vestal from the CPC is directly deposited according to the cob parameters that are set at the input of the control system. Among the main parameters that directly affect the quality of engraving, as a rule, the following parameters should be distinguished [23]:

  The number (density) of engraving lines, which depends on the quality of the original image;
  Laser power, which determines the choice of material for engraving and the maximum engraving depth;
  The speed of the laser moving along the engraving surface and the engraving step determined by the type of Stepper Motors (SM) and the resolution of the laser beam, which depends on the type of the selected laser module, resulting in the need to configure the laser machine with CNC to ensure the quality of the image, represented as a “gray” box [24], with a vector of input parameters *x*_1,_*x*_2,_*x*_3,_*x*_4,_ and *x*_5_, and the vector of output parameters *y*_1,_*y*_2,_ and *y*_3,_ as shown in Figure 1.

The vector of input parameters includes the number of image lines (lines/mm), power and speed of the laser (Watt), engraving step (mm), and laser beam resolution (dpi):

(1) $$\begin{array}{c} X=\left\{L,S,V,N,F\right\}, \end{array}$$

where *L* is the number of lines (lines/mm), *S* is the laser power (Watt), *V* is the laser speed (m/s), *N* is the engraving step (mm), and *F* is the laser beam resolution (dpi). Depending on the requirements for the material on which the engraving is to be performed, the parameters *F* and *S* can be changed by installing a more powerful laser module. All other parameters can be changed programmatically through the CNC machine control system.

The vector of output parameters includes the line density per mm, the image engraving time, and the burn depth as shown in the equation as follows:

(2) $$\begin{array}{c} X=\left\{q,t,h,k\right\}, \end{array}$$

where *q* is the line density (line/mm), *t* is the time (s), *h* is the burn depth (mm), and *k* is the image contrast. Note that the image contrast *k* depends on the movement speed of the laser module, which implements SMs, and the accuracy of the model position, which serves as the basis for choosing the type of SM.

The analysis of the relationship between input and output parameters of the control object allows drawing the following conclusions in the presented work:

- Number of lines *L* allows to achieve high-quality images by passing the laser a specified number of times
- The laser power *S* sets its range of work associated with the speed of movement to simulate the color of the image, i.e., the depth of burning *h*
- The laser movement speed *V* depends on the hardware of the engraver, which affects the time of engraving the image *t*
- The image contrast *k* depends on the speed of the laser *V*
- The engraving step *N* depends on the density of the obtained lines *q*, that is, the fill density of the image
- The detailed level of the received image, i.e., the quality of execution of an engraving, depends on the resolution of a laser beam

To create more control through CNC machines, it is necessary to select components such as control boards, SMs, temperature sensor, laser, and actuator (lead screws).

**Figure 2:**
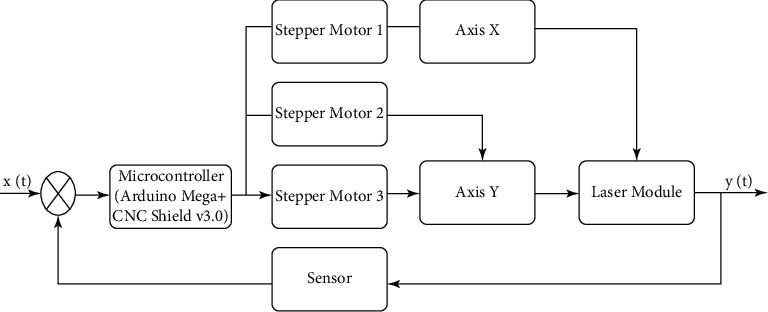
The structure of the control system of the CNC machine.

Based on the analysis of the relationship between the input and output parameters of the portable laser engraver developed, the authors propose the following structure of the cutting system with a laser engraver based on a CPC, as shown in Figure 2.

The input parameters are denoted by *x*(*t*), which is a file in Bitmap Picture format (∗.bmp). The file ∗.bmp formatted file can be loaded into the control system, such as in microcontroller ATmega2560 [25], installed CNC Shield v.3.0 [26], and A4988 drivers [27], where 3-SMs are also connected to the board. SM 1 is responsible for moving along the *X* axis (Axis *X*), and SM 2 and 3 are responsible for moving along the *Y* axis (Axis *Y*). The use of two SMs allows ensuring the accuracy of moving the laser module along the machine frame without using a more expensive SM with two shafts, resulting in reducing the cost of the experimental layout. The proposed solution allows moving the laser in Cartesian coordinates and controlling the engraving working area using the feedback through the Sensor as shown in Figure 2.

Figure 2 illustrates the way to select the elements of the machine control system with CNC, namely the microcontroller, SMs, actuators, and laser.

**Figure 3:**
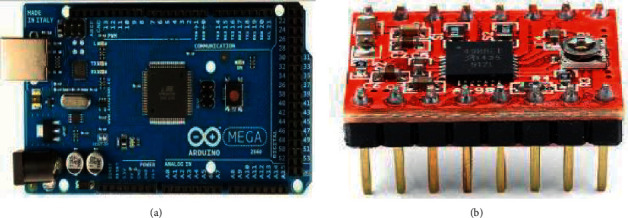
(a) Control board is Arduino Mega 2560-M16U [26]. (b) A4988 SM driver [27].

The selected control board in the proposed work is Arduino Mega 2560-M16U as shown in Figure 3(a), which has a low cost, simple instruction, and easy to provide a good interface and compatibility with operating systems, such as Windows, Macintosh OSX, and Linux, taking into consideration that most of the operating systems are focused only on Windows [28].

The selection of the Arduino Mega 2560 control board requires choosing an expansion board that reduces the number of wires and facilitates the installation. Drivers for the SMs are installed on the expansion board as in Figure 3(b), where jumpers for dividing the motor pitch are installed, which further affects the image clarity parameter.

**Figure 4:**
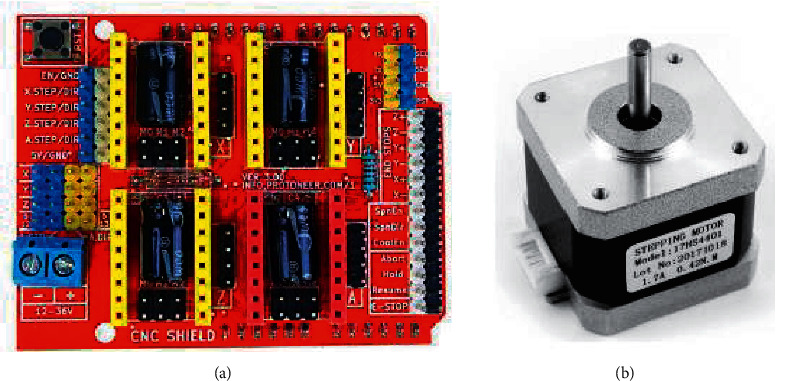
(a) CNC shield extension board v3.0 [26]. (b) Stepper mottor nema 17 [29].

CNC Shield v3.0 [26] was chosen as the expansion board, which is a specialized board for working with CNC machines as shown in Figure 4(a).

Given that this small-bore portable laser engraver is being developed to be used in ARSS, the main condition is high maintainability and availability. Based on this, the authors believe that in the field, if one of the elements of the control system fails, it is possible to make an emergency replacement in the PnP (Plug and Play) mode. Therefore, the proposed hardware modules for the implementation of the control system are the best solution, in contrast to all the hardware elements of the control system integrated into one board.

Next, the SM is chosen, which converts electrical voltage pulses of the control voltage into discrete angular or linear movements of the rotor with its potential fixation in the desired positions. SM allows moving the functional tool throughout the working area of the machine with CNC. The functional tool is regarded as one of the main parts of the machine because it is responsible for the main operation, and where the CNC is intended for the surface treatment of the workpiece, or other operation. The hybrid Stepper Mottor Nema 17 (17HS4401), which is shown in Figure 4(b), is used for the control task of *X* and *Y* axis movement [29].

**Figure 5:**
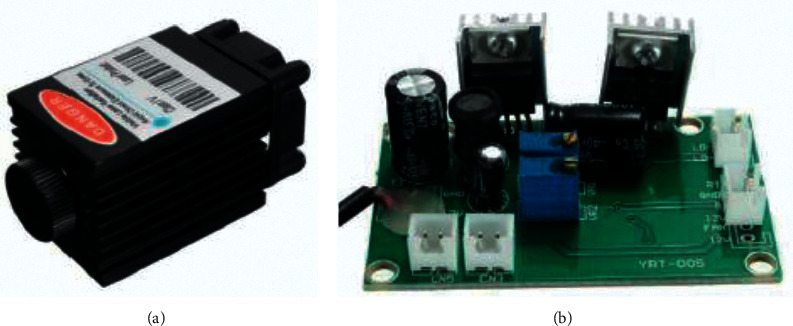
Focusable 500 mw diode laser and driver board [31]. (а) Low-power diode laser; (b) laser driver board.

Powerful lasers are used on industrial CNC machines, the most common of which are CO_2_ lasers and fiber lasers [30]. Such lasers require expensive maintenance. To decrease the maintenance cost, a low-power diode laser shown in Figure 5(a) and a laser driver board shown in Figure 5(b) [31] were chosen in the proposed work because it is easy to operate and has relatively high performance characteristics. Moreover, on the laser driver board, there is a TTL channel that monitors the power, where the laser is tuned off (zero power) at 5 V and tuned on (full power) at 0 V. TTL enables controlling a few parameters as the shades of gray, power of the laser module 1.6 W, wavelength 445–450 nm, color blue, supply voltage DC 12 V, and Frequency TTL from 0–20 kHz.

Comparisons of the studied laser parameters for small machines are presented in Table 1.

As can be seen from Table 1, the selected laser at an output power of 500 mW has different wavelengths and prices. Considering that a laser is a module with a life cycle of 3,000 to 8,000 hours of operation, the authors of the experimental machine with CNC under development chose the Focusable 500 mW, laser model.

### 2.2. The CNC Machine Control System and the Generalized Main Parameters

According to the selected control system elements of the CNC machine and the developed block diagram proposed in Figure 2, it is further necessary to synthesize the control system for each element. To do so, it is proposed to use the theory of automated control, where all the selected elements in the form of transfer functions are presented in this paper. The transfer function is one of the ways to mathematically describe a dynamic system. In automatic control theory, the transfer function of a continuous system is the ratio of the Laplace transform of the output signal to the Laplace transform of the input signal at zero initial conditions [35]. Accordantly, the developed control block diagram of the workbench with the CPC is described mathematically for each element through the transfer function [36].

The transmitter transfer function equation is
  (3) $$\begin{array}{c} W_{\text{sen}}=\frac{0.4412}{0.02s^{2}+s}. \end{array}$$
The transfer function of the first engine equation is
  (4) $$\begin{array}{c} W_{d1}=\frac{1}{0.1s+1}. \end{array}$$
The transfer function of the second engine equation is
  (5) $$\begin{array}{c} W_{d2}=\frac{1}{4.49s+1}. \end{array}$$
The transfer function of the third engine equation is
  (6) $$\begin{array}{c} W_{d3}=\frac{31.62}{0.4732s+1}. \end{array}$$
The transfer function of the laser module equation is
  (7) $$\begin{array}{c} W_{l}=\frac{4.49s+1}{0.000398s^{2}+0.01995s+1}. \end{array}$$
The transfer function of the first shaft equation is
  (8) $$\begin{array}{c} W_{sh1}=0.0335s+1. \end{array}$$
The transfer function of the second shaft equation is
  (9) $$\begin{array}{c} W_{sh2}=0.01s+1. \end{array}$$

**Figure 6:**
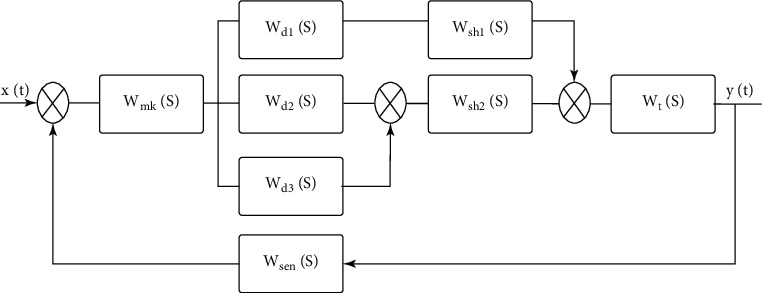
Structure of the automated control system of the CNC machine.

Based on the developed structure presented in Figure 2 and the transfer functions shown in equations (3)–(9), the control system can be represented as the structure of the transfer functions, as illustrated in Figure 6.

The next step is to calculate the transfer function of the open and closed loop system [36, 37]:

The transfer function of the open loop system is given in the following equation:
  (10) $$\begin{array}{c} W_{р}\left(s\right)=W_{mk}\left(s\right)\times \left(\left(W_{d2}\left(s\right)+W_{d3}\left(s\right)\right)\times W_{sh2}\left(s\right)+W_{d1}\left(s\right)\times W_{sh1}\left(s\right)\right)\times W_{l}\left(s\right)\times W_{sen}\left(s\right). \end{array}$$
The transfer function of the closed-loop system is given in the following equation:
  (11) $$\begin{array}{c} W_{3}\left(s\right)=\frac{W_{mk}\left(s\right)\times \left(\left(W_{d2}\left(s\right)+W_{d3}\left(s\right)\right)\times W_{sh2}\left(s\right)+W_{d1}\left(s\right)\times W_{sh1}\left(s\right)\right)\times W_{l}\left(s\right)}{1+W_{mk}\left(s\right)\times \left(\left(W_{d2}\left(s\right)+W_{d3}\left(s\right)\right)\times W_{sh2}\left(s\right)+W_{d1}\left(s\right)\times W_{sh1}\left(s\right)\right)\times W_{l}\left(s\right)\times W_{sen}\left(s\right)}. \end{array}$$

Making the substitution equations (1)–(7) into equations (8) and (9), the overall transfer function of the open loop system becomes the following equation:

(12) $$\begin{array}{c} W_{p}=\frac{2.098s^{3}+27.72s^{2}+68.71s+13.95}{1.691\times 10^{-6}s^{7}+0.0001902s^{6}+0.01062s^{5}++0.3212s^{4}+2.821s^{3}+5.103s^{2}+s}, \end{array}$$

and of the closed loop system equation as follows:

(13) $$\begin{array}{c} W_{3}\left(s\right)=\frac{2.098s^{3}+27.72s^{2}+68.71s+13.95}{1.691\times 10^{-6}s^{7}+0.0001902s^{6}+0.01062s^{5}+0.3212s^{4}++4.922s^{3}+32.82s^{2}+69.71s+13.95}. \end{array}$$

The next step is to assess the stability of the open loop system at the roots of the characteristic equation. To do so, it is necessary for the transfer function of a closed loop system (13) to select the denominator in the form of a characteristic equation (14) and find its roots equation (15). If all roots of the characteristic equation have the same sign (either all negative or all positive), then the system will be stable. Otherwise, the system will not be stable.

(14) $$\begin{array}{c} 1.691\times 10^{-6}\lambda^{7}+0.0001902\lambda^{6}+0.01062\lambda^{5}+0.3212\lambda^{4}+4.922\lambda^{3}+32.82\lambda^{2}+69.71\lambda +13.95=0, \end{array}$$

(15) $$\begin{array}{c} \left\{\begin{array}{c} \lambda_{1}=-25.3646+34.9229i;\\ \lambda_{2}=-25.3646-34.9229i;\\ \lambda_{3}=-24.0819+3.6019i;\\ \lambda_{4}=-24.0819-3.6019i;\\ \lambda_{5}=-10;\\ \lambda_{6}=-3.3537;\\ \lambda_{7}=-0.2227. \end{array}\right) \end{array}$$

**Figure 7:**
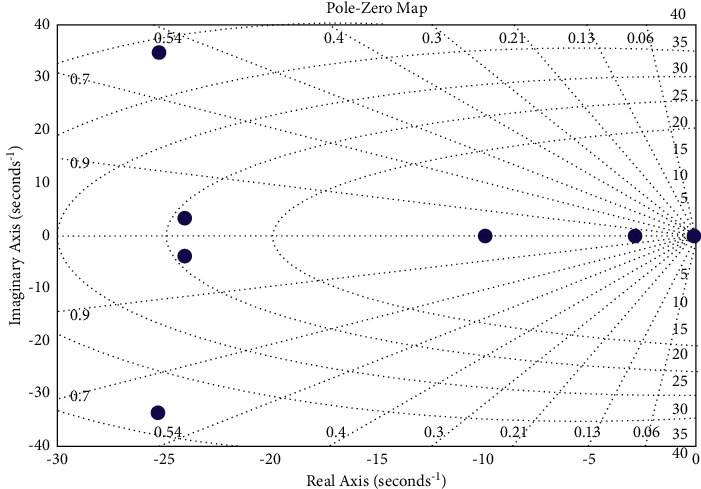
Location of the characteristic equation roots.

Equation (15) shows that the sign of all roots are the same (negative), which means that the system is stable. To plot the location on the complex plane of the roots of the characteristic level equation (15), a package of applications is used to solve the technical calculation challenges by MatLab. Based on the stability conditions for linear automatic control systems, which can be formulated as follows: the system is regarded as stable when all the roots of its characteristic equation are located on the left side of the axis, i.e., all the real parts of the roots are negative, taking into consideration that it is enough to have one real part of any root in the right side of the axis to make the system unstable. As can be seen in Figure 7, the location of characteristic polynomial roots on the complex plane is along the axis *y*, where *y* is the imaginary part, and along the axis *x*, where *x* is the real part. Accordingly, all roots are located on the left side of the axis. Therefore, the system is regarded as stable.

As a result, it is possible to assume that the closed loop system is stable; however, it is required to estimate the stability of the closed-loop system by Mikhailov's criterion to fully evaluate the proposed system, where the Mikhailov criterion is described as follows.

Mikhailov curve vector *D(jw)* tests the system's stability by increasing the frequency from 0 to *∞*, *∞*, where the system is considered stable when it starts moving on a real positive semiaxis and rotates only counterclockwise, passing sequentially *n* quadrants of the coordinate plane, where *n* is the degree of the characteristic polynomial, i.e., the number of the roots of the characteristic polynomial [38].

The Mikhailov curve for stable systems is characterized by having a smooth spiral shape, and its end goes to infinity in that quadrant of the coordinate plane. If, as a result of the calculation, the control system of a portable laser engraver is stable, then the system becomes stable from external disturbances and will return to a state of rest.

**Figure 8:**
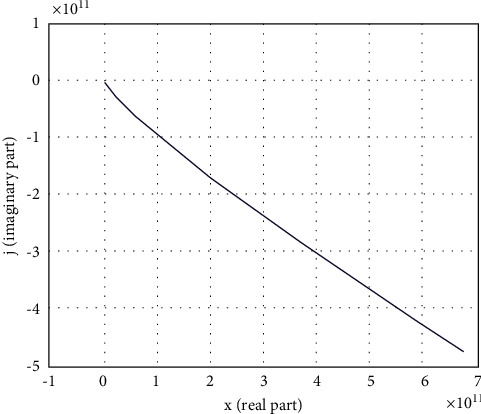
Mikhailov curve for the proposed system with 7 roots of the characteristic polynomial.

Using MatLab, the Mikhailov curve was constructed, which is shown in Figure 8.

**Figure 9:**
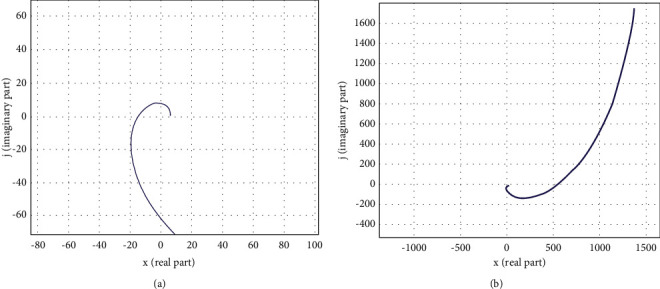
Mikhailov curve at the approximation range. (а) Range one. (b) Range two.

**Figure 10:**
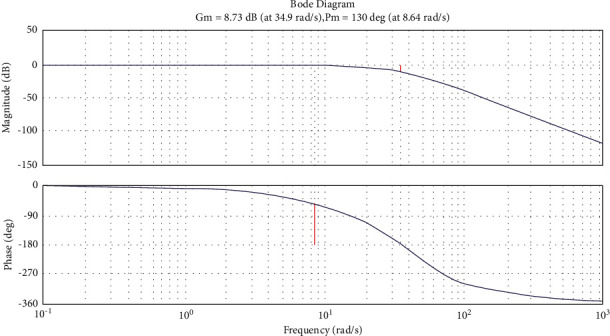
DLFC and AFPFR of CNC control systems.

Though Figure 8 shows that the Mikhailov curve consistently covers 7 quadrants, to obtain clearer curves, the range of the coordinate axes can be changed as shown in Figures9(a) and 10(b).

The graphs of Figures 9(a) and 10(b) show that the Mikhailov curve consistently covers 7 quadrants, which is equal to the degree of the characteristic polynomial without intersecting the origin, and fully meets the requirements of the Mikhailov criterion. Consequently, the closed loop system is regarded as stable.

**Figure 11:**
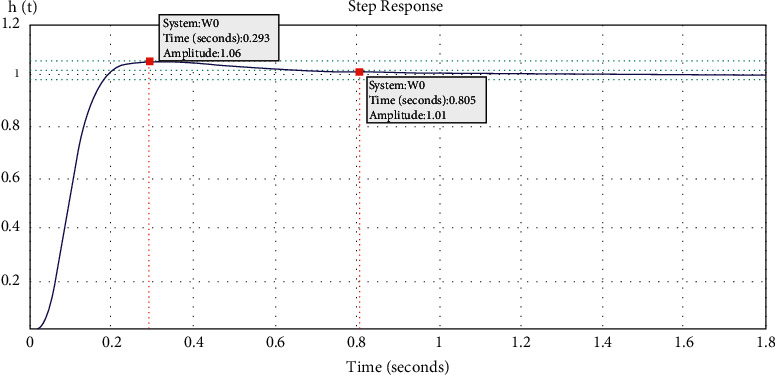
Transient characteristics of the CNC control system.

To define direct indicators of system's quality, the graph of the transient characteristic of the system is obtained and shown in Figure 11.

From Figure 11, the time of the system's regulation is determined, which is equal to *t*_*p*_=0.805*s* , and the value of over-regulation is calculated in the equation as follows:

(16) $$\begin{array}{c} \sigma =\frac{h_{\max}-h_{\infty }}{h_{\infty }}\times 100\%=\frac{1.06-1.01}{1.01}\times 100\%=4.95\%. \end{array}$$

Under the operating conditions, the parameters of the control system vestal with CNC may vary within certain limits (wear, temperature fluctuations, friction, surface angle, etc.), where these fluctuations in parameters can lead to a loss of stability of the system mainly when operates close to the stability limit. Therefore, the designed ACS for CNC should work far from the limit of sustainability. In addition, it is necessary to determine the stability margins of the Westat control system with CNC by the values of the phase and amplitude margins; accordingly, a decibel log frequency characteristic (DLFC) and Logarithmic amplitude-phase frequency response (AFPFR) are obtained and shown in Figure 10.

Figure 10 identifies the following indicators of stability:

- The amplitude stability margin (Δ*А*)=8.73  *dB*
- The phase margin of stability (Δ*ϕ*)=130°

The obtained values of stability indicate that the value of the margin of stability in the amplitude falls in the range of 6–20 dB, whereas the values of the margin of the phase fall in the range of 30°–600°, which indicates that the developed system is normally damped system. Based on the study of the stability of the developed control system of the CNC machine, the quality indicators and the stability margins, it is concluded that the selected elements of the system and its structure meet all the conditions of the design of such systems.

### 2.3. Optimization of CNC Machine Tool Control System

**Figure 12:**
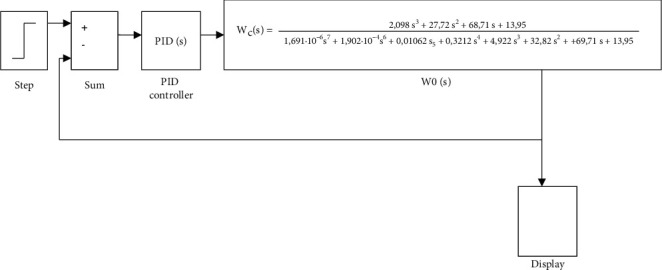
Layout of the CNC machine tool control system with PID controller in MatLab.

CNC laser machine is a complex multidimensional object, and the values of its adjustable and outrageous parameters are dynamic. There are some difficulties in developing a management system for such facilities. For example, in the transfer function of the laser power control area that is applied to obtain the desired color intensity, which is calculated according to known methods of setting standard regulators for this class of objects, accordingly, corrections and, in many cases, nonstationary noise and perturbations acting on objects are required, resulting in considering the automatically adjustment of the control settings or selecting the optimal settings as an urgent task. For such a task, modeling is carried out by firstly building a model of the CNC machine control system with PID controller, as shown in Figure 12.

**Figure 13:**
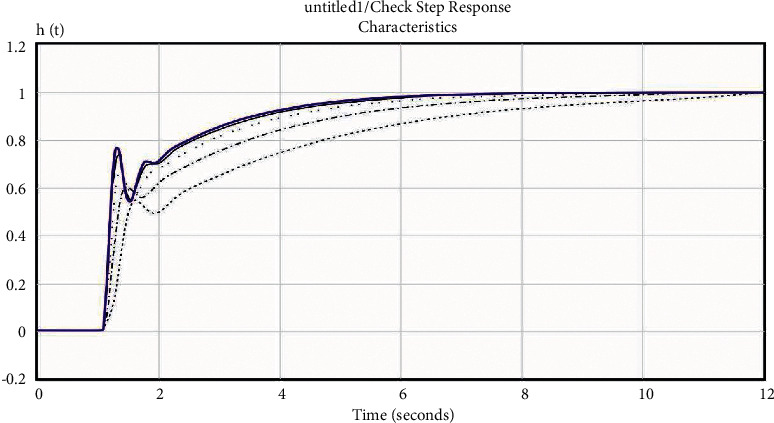
Simulation results of the transient characteristics.

Determining the optimal parameters of the controller is an iterative process, which consists of 21 iterations, in which the parameters of the PID controller were selected and tested experimentally. At the same time, it is noted that the number of iterations depends on the requirements of the laser engraver control system (quality, reliability, accuracy, est.). The results of the optimization are the calculated optimal parameters of the PID controller that is shown in Figure 12. Moreover, the graphs of the calculated transient characteristics are shown in Figure 13.

**Figure 14:**
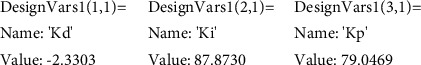
The obtained values of the coefficients of the PID controller.

The obtained results shown in Figures 13 and 14 are not optimal because the parameters of the laser machine system controller are optimized according to certain parameters. To obtain optimal results for the system according to the operation parameters, a block diagram of the model based on Figure 12 is built, and the “Saturation” block is added, which is a nonlinear device that acts as an ideal limiter.

**Figure 15:**
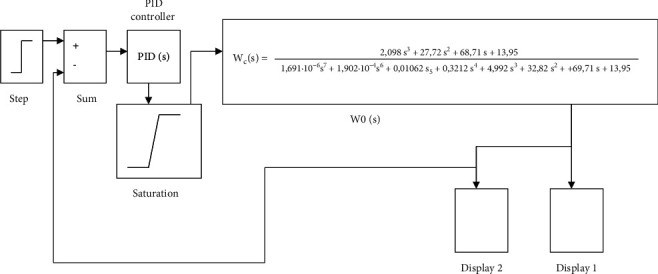
The investigated structural scheme of the model for optimization of the PID controller in MatLab.

The saturation block keeps changing the output signal till it becomes equal to the input signal, which is the adjusted threshold for the upper and lower bounds. When reaching the adjusted thresholds, the output signal stops changing, resulting in improving the PID controller.  Figure 15 shows the block diagram of the optimized model of the PID controller in MatLab.

**Figure 16:**
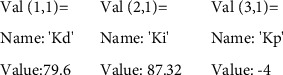
Optimal coefficients of the proposed PID controller.

Based on the simulation results, the PID controller parameters were optimized, and the optimal PID controller coefficients were obtained, which are presented in Figure 16.

The following coefficients were selected for the developed CNC workbench control system: coefficient of integral component = 87.32; coefficient of differential component = 79.6, and the coefficient of the proportional component = −4.

**Figure 17:**
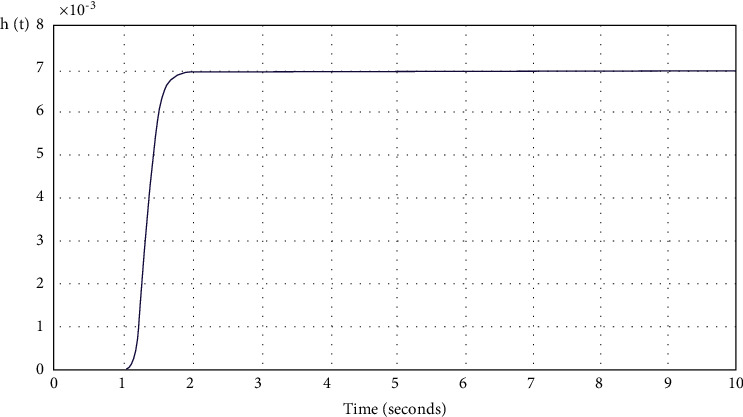
Transition process of CNC control system with optimized parameters UNDER the regulator.

The transition graphs with optimized controller parameters are shown in Figure 17.

## 3. Development of the Structure and Prototype of the Laser Workbench Model with CNC

The full model structure contains hardware structure, parameters setting, and the operating software.

### 3.1. Hardware and Setting Development

**Figure 18:**
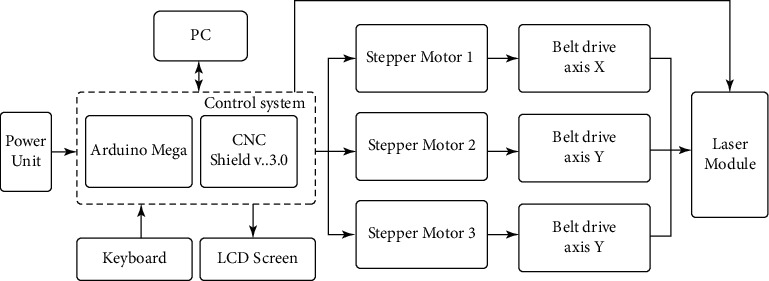
Block diagram of a CNC laser machine.

Based on the proposed block diagram of the machine with CNC (Figure 2) and the selected hardware modules (Figures 3–5), a block diagram was developed, which is presented in Figure 18.

**Figure 19:**
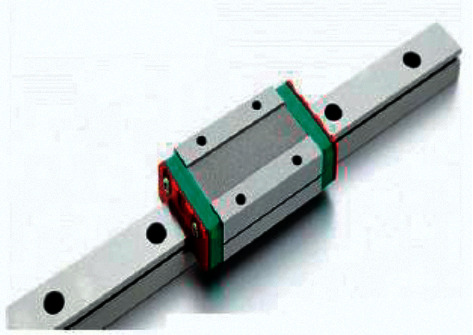
Rail linear guide [39].

Accuracy is important for CNC, so the design with the use of profile and rails allows for achieving the highest possible accuracy, which determines the scope of the application. The surface of the rail is pretreated and carefully sanded to avoid the slightest corrosion and dents. Due to the presence of side recesses in the form of a holder for balls, the carriage moves along the beam. Unlike a round shaft, the collision area is not a point but a line. This creates certain advantages, such as increasing the wear resistance of the rail, increasing the accuracy of the machine, increasing the load capacity, and improving the ability to withstand loads. Accordingly, the minimum backlash or its complete absence is provided. To achieve maximum accuracy in the developed model of the CNC machine, it is proposed to use rail linear guides as shown in Figure 19 [39].

**Figure 20:**
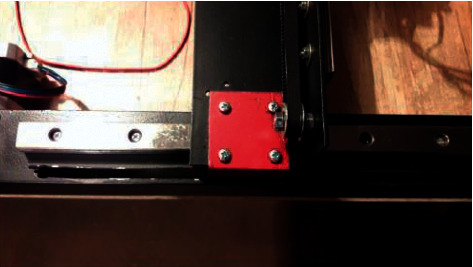
Attaching the portal to the *Y* axis.

A SM is attached to the aluminum so that its shaft is inside the profile, and the guide is fixed from above by the cut hole from the corner that will fasten the portal with the *Y* axis. The portal is mounted on the carriage, the operation is symmetrical, and the belt is fastened inside the profile.  Figure 20 shows the mounting of the portal with the *Y* axis.

**Figure 21:**
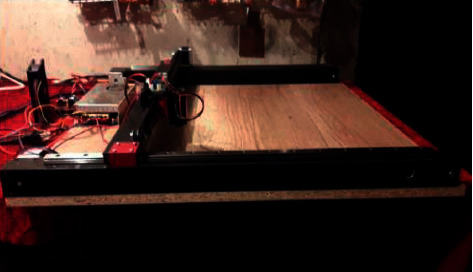
View of the CNC machine from the side.

After that, the design is fastened on the chipboard, the power is supplied, and hence the laser is fastened on a portal carriage.  Figure 21 shows a side view.

**Figure 22:**
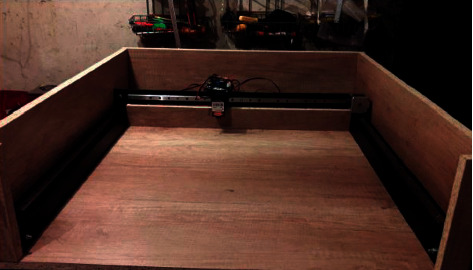
The assembled layout prototype of the Laser machine with CNC.

All electronic parts are placed in the rear and covered with a piece of chipboard resulting in building the body as shown in Figure 22, which shows the model of the assembled CNC laser machine.

After assembling the prototype of the layout, the voltage on the driver board A4988 is adjusted for CD, and finally, the value of the step (Step) of the belt drive is calculated.

The correct current setting for the SM affects the following characteristics: noise reduction from the operation of the machine with CNC, getting rid of missing SM steps, and reducing the heat of the SM or driver.

To calculate the voltage on the A4988 driver board, the following formula is used [40]:

(17) $$\begin{array}{c} V_{\text{ref}}=I_{\max}\times 8\times R_{s}, \end{array}$$

where *V*_*ref*_ is the voltage on the driver board (*v*), *I*_max_ is the rated motor current (*A*), and *R*_*s*_ is the resistance of the resistor on the board A4988 (0.1 *Ω*).

Due to the fact that the operating current of the motor is equal to 70% of the holding current, the obtained value of 1.36 V (from cutout 15) must be multiplied by 0.7. Otherwise, the temperature of SM in the hold mode increases resulting in heating the SM. Accordingly, equation (17) is modified as shown in the following equation:

(18) $$\begin{array}{c} V_{\text{ref}}=1.36\times 0.7=0.952. \end{array}$$

**Figure 23:**
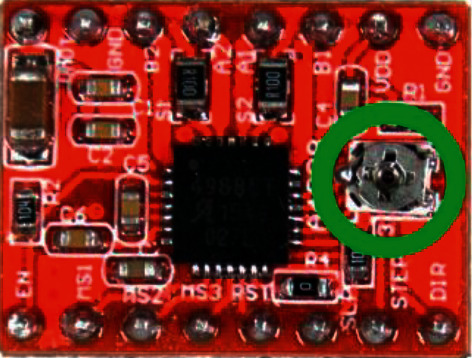
The variable resistor on the A4988 board.

The result of 0.952 V should be adjusted on the board by adjusting the variable resistor shown in Figure 23.

To “fine tune” the A4988 driver, you need a 5 V voltage source (Arduino Nano controller). You will also need a voltmeter and a small screwdriver. First, we connect the common wire of the Arduino Nano to the common driver and +5 V to the power output of the driver logic called VDD. We power the Arduino Nano and use a voltmeter to measure the voltage between the common wire and the trimmer motor (in the green circle in Figure 23). Next, twisting the construction resistor with a screwdriver, we achieve the calculated voltage of 0.952 V on the voltmeter. The specifics of setting the A4988 driver is that during the setting, it is necessary to carry out without contact of the hand to the metal part of the screwdriver since the hand can act on the measured electrical circuit. This, accordingly, can introduce errors into the settings and, consequently, into the quality of the image obtained by laser engraving. If the voltage does not change when the resistor slider rotates, then the A4988 driver chip is out of order.

To calculate the value of the step (Step) for the belt drive, it is required at first to determine the number of the needed steps to enable the engine's shaft to generate one full revolution, i.e., 360°, so, to obtain 200 steps for example, the single step adjusted to rotate the shaft by 1.8° according to the following formula:

(19) $$\begin{array}{c} N_{d}=\frac{360}{1.8}=200, \end{array}$$

where *N*_*d*_ is the number of the steps needed to obtain one full rotation for the shaft.

Therefore, it takes 200 steps (pulses) to make a full shaft rotation. Moreover, the A4988 driver enables increasing the number of engine steps by controlling the intermediate steps, which increases the accuracy of the CNC machine by reducing torque. To adjust the step on this driver, applying the voltage to certain foams according to Table 2 is required.

Choose the largest division of step 1/16 and install jumpers on the expansion board CNC Shield v3.0 where the A4988 driver board is located.

For example, calculating the number of steps at the full rotation of the motor shaft with the division of step 1/16 can be obtained according to equations (19) and (20):

(20) $$\begin{array}{c} N_{d}=\frac{360}{1.8\times 1.6}=3200. \end{array}$$

Based on the results of the calculation, it is possible to determine the value of the step for the belt drive according to the formula as follows:

(21) $$\begin{array}{c} N=\frac{\left(N_{d}/n\right)}{N_{\text{step}}}, \end{array}$$

where *N* is the number of the step for the belt drive, *n* is the number of gear teeth (GT2 toothed belt with 2 mm pitch installed), and *N*_step_ is the gear pitch frequency. For the developed layout, the value of the step for the belt drive is 80 (*N* = 80).

### 3.2. Firmware and Configuration of the CNC SHIELD V3.0 Workbench Software

**Figure 24:**
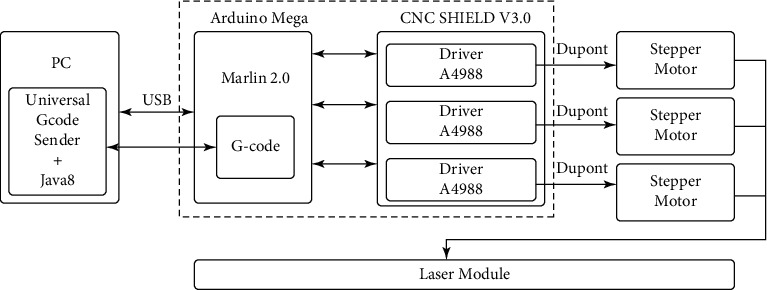
Consolidation of the information model of the relationship of the software and the hardware modules of the workbench with CNC.

Based on the selected hardware modules (Figures 3–5) of the developed laser workbench with CNC and block diagrams (Figure 18), the next step is to develop an information model of software relationships.  Figure 24 shows the aggregation of the information model of the relationship between the software and the hardware modules of the workbench with CNC.

In the first step, download Marlin 2.0 Firmware as the most optimized for CNC machines [41]. Open the file X:\∗∗∗\Marlin-2.0.x\Marlin.ino in the Arduino IDE. Reconfigure the configuration.h file. For work with the developed laser machine with CNC. First of all, adjusting the speed of the Serial Port to work with the Arduino Mega 2560-M16U is required, which does not need to change:

# *define BAUDRATE 250000 на # define BAUDRATE 115200*.

Next, the board to which the laser control module is connected is required to be specified, which depends on the used foam. In the proposed work, the following was specified:

# ifndef MOTHERBOARD

# define MOTHERBOARD BOARD_RAMPS_14_EFB

# endif

The next step is to specify the used laser, where it is not necessary to specify the same settings:

# define EXTRUDERS 1

After that, it is necessary to adjust the operation of thermistors used to measure the table and extruder. As the proposed work is for a laser engraver, the firmware must indicate their absence (Appendix A).

Adjustment of the PID controller is carried out in the following parts of the firmware, where it is needed to specify the calculated values:

# define DEFAULT_bedKp 79.6

# define DEFAULT_bedKi 87.32

# define DEFAULT_bedKd-4

Next, it is needed to spend the mood of the limbs, in the form of maximum and minimum position, and in which direction they should park, which is very important because it depends on the grid of the CNC laser machine.

# define USE_XMIN_PLUG

# define USE_YMIN_PLUG

# define USE_ZMIN_PLUG

The next step is to specify the logic of the suns, normally open or normally closed. This is done in the following lines of code (Appendix B).

Next, it must be indicated which drivers of SMs are used in the assembled machine:

# define X_DRIVER_TYPE A4988

# define Y_DRIVER_TYPE A4988

# define Z_DRIVER_TYPE A4988

Now it is necessary to adjust the “steps,” i.e., how far the carriage of the laser engraver will travel along the axis. The distance depends on the belt, the driver of the soft engine, the engine itself, or the nut screw. To calculate the steppes according to (18-19).

#define DEFAULT_AXIS_STEPS_PER_UNIT {80, 80, 400, 500}

Then the speed and acceleration are set, as shown in Appendix C:

The next step is to adjust the direction of SMs rotation, if they work properly, except for the second motor, so it must be inverted:

define INVERT_X_DIR false

# define INVERT_Y_DIR true

# define INVERT_Z_DIR false

The last step is to specify the direction of parking and the size of the work area, as shown in Appendix D.

After configuring the laser engraver firmware, it is needed to save the Marlin 2.0 Firmware and compile it for testing.

## 4. Examples of Trial Engraving as Confirmation of the Performance of the Laser Engraver Layout

To verify the accuracy of the decisions made, which hardware and software were made for trial engraving on the developed model of the laser engraver.

**Figure 25:**
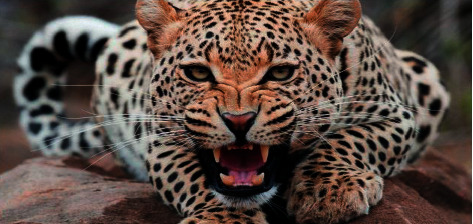
Test image.

**Figure 26:**
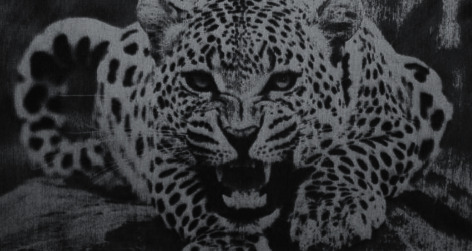
Results of test image (Figure 25).

In Figures 25 and 26, sample drawings for engraving are illustrated for two different engraving surface materials.

Figure 26 shows the results of engraving of Figure 25 on the engraving surface of plywood 4 mm, where the engraving time is 2 hours 30 minutes. The obtained results of the proposed first example show that the accuracy of the resulting image is regarded as high.

Figure 26 shows the results of engraving, where the high engraving precision is clear. However, despite the high accuracy of engraving for the example in Figure 26, it should be noted that for a more detailed assessment of the quality and accuracy of engraving, it is necessary to conduct a number of test experiments, taking into consideration that such experiments and their full description are beyond the scope of this article, moreover, implementing the deep learning technique can lead to much clearer results such as implementing the deep learning in [42]; however, this is regarded as the future work.

It is important to note that the layout of the laser engraver presented in this work showed its fundamental capabilities for engraving on different media, overall performance, compliance with the theoretical foundations, and practical implementation of the laser engraver proposed in the experimental sample. The conducted experiments confirm that the developed control system based on the PID controller allows high-quality engraving on leather, plywood, and wood. In this aspect, the key is the choice of optimal control parameters for controlling the operation of a laser engraver.

Based on the above, the main goal of this study has been achieved, and an experimental portable laser engraver has been developed and assembled, which has shown its performance and engraving quality.

## 5. Conclusions

The proposed work in this article is focused on designing and implementing a laser engraving system based on modern industrial Computer Numerical Control (CNC), based machines which shows the great complexity of their application in Armament Repair Shop Set (ARSS) systems. This is due to the large overall dimensions, limited mobility, and the complexity of repairs in the field. The authors made an assumption about the development of small-sized portable laser engravers based on CNC. There is also the possibility of future mobile adaptation to a CNC milling machine by replacing the working body, which is expected to save a lot of space for other equipment. Based on this solution, the authors set a number of tasks, during the solution of which an analysis was made of the relationship between the input and output parameters of the control object vectors. To comply with these relationships, the authors proposed a block diagram of small-sized portable CNC-based laser engravers. Moreover, the authors developed and mathematically described the control system for selected modules, which applies the theories of automated control. The construction and synthesis of the designed system in MatLab were carried out, and it was proved that the developed system is stable. To ensure the quality and accuracy of laser engraving, the authors optimized the developed system by including a PID controller in it. To check the correctness of the decisions made and the mathematical conclusions, the authors developed an experimental prototype of a small-sized portable CNC-based laser engraver.

In the course of the study, the authors optimized the synthesized control system by using a PID controller in it. By means of mathematical modeling, the indicators of the PID controller were obtained, which accordingly increased the stability of the control system to the occurrence of external vibrations and parasitic factors that could adversely affect the quality of laser engraving.

Finally, the authors applied the proposed model to several actual images, and the obtained results are regarded as high in quality, though just 2 hours and 30 minutes were required to finish the whole laser engraving process in such a small area on different types of surfaces, which practically proves the validity and efficiency of the proposed mode.

The future work is planned to conduct a detailed assessment of the quality and accuracy of engraving based on the developed layout. It is also planned to modify the structures to develop a universal CNC machine with a replaceable working body laser engraver/CNC milling machine.

## Figures and Tables

**Table 1:** Comparison of the parameters of the studied lasers.

Laser model	Wave-length (nM)	Output power (mW)	Laser shape	Reverse polarity protection	Warm-up time	Price ($)
Focusable 500 mw [31]	808	500	Dot (rectangular or oval)	+	0	26
405MD-500 [32]	405	500	Dot (oval)	+	0	42
SRBI FB04-500 [33]	405	500	Dot (rectangular or oval)	+	0	41
FB03-500 [34]	405	500	Dot (oval)	+	0	62

**Table 2:** Step settings on the A4988 driver.

MS0	MS1	MS2	Step resolution
Low	Low	Low	Full step
High	Low	Low	1/2
Low	High	Low	1/4
High	High	Low	1/8
High	High	High	1/16

## Data Availability

We confirm that the data used in this work is available at and ready upon request. Please contact the 4^th^ author (e-mail: viacheslav.liashenko@nure.ua).

## Appendix

### A. Operational Adjustment of the Thermistors

#define TEMP_SENSOR_0 0.ik

#define TEMP_SENSOR_1 0.

#define TEMP_SENSOR_2 0.

#define TEMP_SENSOR_3 0.

#define TEMP_SENSOR_4 0.

#define TEMP_SENSOR_5 0.

#define TEMP_SENSOR_6 0.

#define TEMP_SENSOR_7 0.

#define TEMP_SENSOR_BED 0.

#define TEMP_SENSOR_PROBE 0.

#define TEMP_SENSOR_CHAMBER 0.

#define TEMP_SENSOR_COOLER 0.

#define TEMP_SENSOR_REDUNDANT 0.

### B. Setting the Sums' Logic at Normally Open or Normally Closed

#define X_MIN_ENDSTOP_INVERTING false.

#define Y_MIN_ENDSTOP_INVERTING false.

#define Z_MIN_ENDSTOP_INVERTING false.

#define I_MIN_ENDSTOP_INVERTING false.

#define J_MIN_ENDSTOP_INVERTING false.

#define K_MIN_ENDSTOP_INVERTING false.

#define X_MAX_ENDSTOP_INVERTING false.

#define Y_MAX_ENDSTOP_INVERTING false.

#define Z_MAX_ENDSTOP_INVERTING false.

#define I_MAX_ENDSTOP_INVERTING false.

#define J_MAX_ENDSTOP_INVERTING false.

#define K_MAX_ENDSTOP_INVERTING false.

#define Z_MIN_PROBE_ENDSTOP_INVERTING false.

### C. Setting of the Speed and Acceleration

#define DEFAULT_MAX_FEEDRATE { 300, 300, 5, 25 }

//#define LIMITED_MAX_FR_EDITING.

#if ENABLED(LIMITED_MAX_FR_EDITING).

#define MAX_FEEDRATE_EDIT_VALUES { 600, 600, 10, 50 }

#endif

#define DEFAULT_MAX_ACCELERATION { 3000, 3000, 100, 10000 }

### D. Setting the Direction of the Park and the Size of the Work Area

#define X_HOME_DIR -1.

#define Y_HOME_DIR -1.

#define Z_HOME_DIR -1.

#define X_BED_SIZE 500.

#define Y_BED_SIZE 500.

#define X_MIN_POS 0.

#define Y_MIN_POS 0.

#define Z_MIN_POS 0.

#define X_MAX_POS X_BED_SIZE.

#define Y_MAX_POS Y_BED_SIZE.
